# Secrecy Capacity Region of the AWGN MAC with External Eavesdropper and Feedback

**DOI:** 10.3390/e25091339

**Published:** 2023-09-15

**Authors:** Haoheng Yuan, Guangfen Xie, Bin Dai

**Affiliations:** 1 School of Information Science and Technology, Southwest Jiaotong University, Chengdu 611756, China; yhhcd@my.swjtu.edu.cn (H.Y.); gfxie@my.swjtu.edu.cn (G.X.); 2 Peng Cheng Laboratory, Shenzhen 518055, China

**Keywords:** AWGN MAC, feedback, secrecy capacity, wiretap channel

## Abstract

For the point-to-point additive white Gaussian noise (AWGN) channel with an eavesdropper and feedback, it has already been shown that the secrecy capacity can be achieved by a secret key-based feedback scheme, where the channel feedback is used for secret sharing, and then encrypting the transmitted message by the shared key. By secret sharing, any capacity-achieving coding scheme for the AWGN channel without feedback can be secure by itself, which indicates that the capacity of the same model without the secrecy constraint also affords an achievable secrecy rate to the AWGN channel with an eavesdropper and feedback. Then it is natural to ask: is the secret key-based feedback scheme still the optimal scheme for the AWGN multiple-access channel (MAC) with an external eavesdropper and channel feedback (AWGN-MAC-E-CF), namely, achieving the secrecy capacity region of the AWGN-MAC-E-CF? In this paper, we show that the answer to the aforementioned question is no, and propose the optimal feedback coding scheme for the AWGN-MAC-E-CF, which combines an existing linear feedback scheme for the AWGN MAC with feedback and the secret key scheme in the literature. This paper provides a way to find optimal coding schemes for AWGN multi-user channels in the presence of an external eavesdropper and channel feedback.

## 1. Introduction

The model of the wiretap channel lays the foundation of physical layer security (PLS). In references [1,2], it has been shown that the secrecy capacity of the additive white Gaussian noise (AWGN) wiretap channel model, which is the maximum transmission rate under the perfect weak secrecy (PWS) constraint, is equal to the difference between the channel capacities of the legal receiver and the eavesdropper, and this indicates that to achieve secrecy, the loss of transmission rate is inevitable.

Though channel feedback does not increase the capacity of a point-to-point memoryless channel [3], references [4,5] found that the feedback channel can be used to generate a secret key shared between the legal parties. Then, the transmitter encrypting via the transmitted message by this key, the secrecy capacity of the wiretap channel can be enhanced. Subsequently, references [6,7] further showed that for modulo-additive and AWGN cases, the secret key schemes in references [4,5] are optimal and achieve the capacities of the same models without feedback and the secrecy constraint. Then it is natural to ask: is the secret key-based feedback scheme still the optimal scheme for the AWGN multiple-access channel (MAC) with an external eavesdropper and channel feedback (AWGN-MAC-E-CF), namely, achieving the secrecy capacity region of the AWGN-MAC-E-CF? The answer to the aforementioned question is no, and this is due to the fact that feedback *increases* the capacity region of the AWGN MAC [8], and the secret key scheme only achieves the capacity region of the AWGN MAC without feedback. Then another question is: what is the optimal feedback scheme for the AWGN-MAC-E-CF, and is the secrecy capacity region of the AWGN-MAC-E-CF equal to the capacity region of the AWGN MAC with feedback, which is in parallel to the fact that the secrecy capacity of the point-to-point AWGN channel with an eavesdropper and feedback equals the capacity of the same model without the secrecy constraint [6,7].

In [9], it has been shown that the classical Schalkwijk-Kailath (SK) scheme [10], which is a capacity-achieving scheme for the point-to-point AWGN channel with feedback, also achieves the secrecy capacity of the point-to-point AWGN channel with an eavesdropper and feedback. Motivated by [9], in this paper, we combine Ozarow’s SK-type scheme for the AWGN MAC with feedback [8] and the secret key scheme in the literature to show that the secrecy capacity region of the AWGN-MAC-E-CF is equal to the capacity region of the AWGN MAC with feedback. The basic intuition behind this scheme is explained below. In [9], it has been shown that the SK scheme satisfies the PWS by itself and achieves the capacity of the point-to-point AWGN channel with feedback. In a similar way, we show that Ozarow’s extended SK scheme [8] satisfies the PWS by itself, however, we find that this SK-type scheme does not achieve the entire capacity region of the AWGN MAC with feedback. To show that every point in the capacity region of the AWGN MAC with feedback satisfies PWS, we split the transmitted message of one transmitter into two parts, where one part together with the message of the other transmitter are encoded by Ozarow’s SK-type scheme, and the other part is encrypted by a key which is generated by the channel noise at the first time instant and this key is only known by the legal parties. Following the security property of the SK-type scheme and the secret key, we show that every point in the capacity region of the AWGN MAC with feedback satisfies PWS, which indicates that the secrecy capacity region of the AWGN-MAC-E-CF is equal to the capacity region of the AWGN MAC with feedback.

## 2. Model Formulation and Main Result

### 2.1. Model Formulation

**Figure 1:**
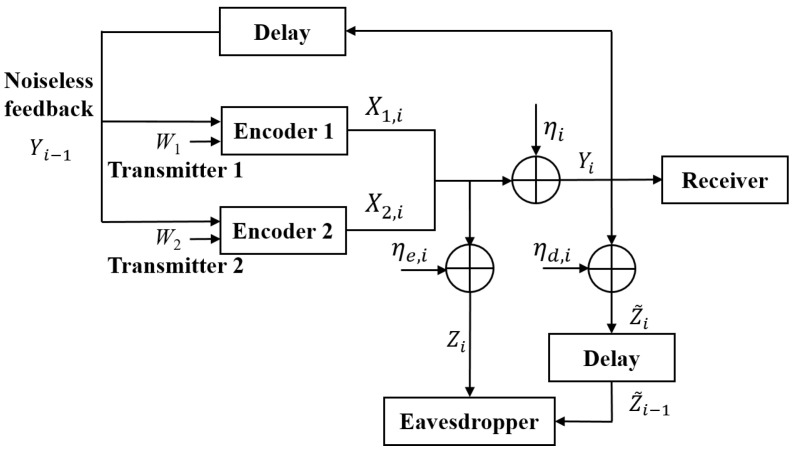
The AWGN MAC with an external eavesdropper and channel feedback.

For the AWGN-MAC-E-CF (see Figure 1), the *i*-th (i∈{1,2,…,N}) channel inputs and outputs are given by

(1) $$\begin{array}{cc} \; & Y_{i}=X_{1,i}+X_{2,i}+\eta_{i},\;\;\;Z_{i}=X_{1,i}+X_{2,i}+\eta_{e,i},\;\;\;i\in \left\{1,2,\ldots ,N\right\}\\ \; & {\tilde{Z}}_{i-1}=Y_{i-1}+\eta_{d,i-1},\;\;\;i\in \left\{1,2,\ldots ,N-1\right\} \end{array}$$

where $X_{k,i}$ (k∈{1,2}) is the channel codeword subject to an average power constraint $P_{k}$, namely, $\frac{1}{N}\sum_{i=1}^{N}E\left[X_{k,i}^{2}\right]\leq P_{k}$, $Y_{i}$ is the legal receiver’s channel output. Here, note that the eavesdropper eavesdrops the codewords $X_{1,i}$ and $X_{2,i}$ by an eavesdropping channel with output $Z_{i}$, and eavesdrops the feedback signal $Y_{i-1}$ by another eavesdropping channel with output ${\tilde{Z}}_{i-1}$. In addition, $\eta_{i}∼N\left(0,\sigma^{2}\right)$, $\eta_{e,i}∼N\left(0,\sigma_{e}^{2}\right)$, $\eta_{d,i}∼N\left(0,\sigma_{d}^{2}\right)$ are AWGNs, and they are independent of one another.

The transmitted message $W_{k}$ (k∈{1,2}) is uniformly drawn in $W_{k}=\left\{1,2,\ldots ,\right|W_{k}\left|\right\}$), and at time i∈{1,2,…,N}, the codeword $X_{k,i}$ (k∈{1,2}) is a (stochastic) function of the message $W_{k}$ and the feedback $Y^{i-1}=\left(Y_{1},\ldots ,Y_{i-1}\right)$. At time *N*, the legal receiver obtains $\left({\hat{W}}_{1},{\hat{W}}_{2}\right)=\psi \left(Y^{N}\right)$, where ψ is the decoding function and the average decoding error probability is denoted by

(2) $$\begin{array}{cc} \; & P_{e}=Pr\left[\left({\hat{W}}_{1},{\hat{W}}_{2}\right)\neq \left(W_{1},W_{2}\right)\right]=\frac{\sum_{w_{1},w_{2}\in W_{1},W_{2}}Pr\left.\{\psi \left(Y^{N}\right)\neq \left(w_{1},w_{2}\right)|\left(W_{1},W_{2}\right)=\left(w_{1},w_{2}\right)\right.\}}{|W_{1}\left|\right|W_{2}|}. \end{array}$$

The eavesdropper’s equivocation rate of $W_{1}$ and $W_{2}$ is denoted by

(3) $$\Delta =\frac{1}{N}H\left(W_{1},W_{2}|Z^{N},{\tilde{Z}}^{N-1}\right).$$

A rate pair $\left(R_{1},R_{2}\right)$ is achievable with PWS if for any ϵ>0 and sufficiently large *N*, there exist channel encoders-decoders such that

(4) $$\frac{\log|W_{k}|}{N}\geq R_{k}-\epsilon ,\;\;\;\Delta \geq R_{1}+R_{2}-\epsilon ,\;\;\;P_{e}\leq \epsilon .$$

The secrecy capacity region $C_{s,mac}^{f}$ of the AWGN-MAC-E-CF is composed of all achievable secrecy rate pairs defined above.

### 2.2. Main Result

The following Theorem 1 shows that $C_{s,mac}^{f}$ equals the capacity of AWGN MAC with feedback.

**Theorem 1.**  *$C_{s,mac}^{f}=C_{mac}^{f}$, where $C_{mac}^{f}$ is the capacity region of the AWGN MAC with feedback [8] (the model of Figure 1 without the secrecy constraint), and it is given by $C_{mac}^{f}={⋃}_{0\leq \rho \leq 1}R\left(\rho \right)$, and*

(5) $$\begin{array}{cc} R\left(\rho \right)=\left\{\left(R_{1},R_{2}\right):R_{1}\leq \frac{1}{2}\log\left[1+\frac{P_{1}}{\sigma^{2}}\left(1-\rho^{2}\right)\right],\right. & \\ R_{2}\leq \frac{1}{2}\log\left[1+\frac{P_{2}}{\sigma^{2}}\left(1-\rho^{2}\right)\right], & \\ \left.R_{1}+R_{2}\leq \frac{1}{2}\log\left(1+\frac{P_{1}+P_{2}+2\rho \sqrt{P_{1}P_{2}}}{\sigma^{2}}\right)\right\} & . \end{array}$$

**Proof.**  See Section 3. □

**Remark 1.**  *Applying the secret key feedback schemes in [6,7] to AWGN MAC with feedback, it is easy to see that any capacity-achieving coding scheme for the AWGN MAC without feedback is secure by itself, which indicates that the secret key inner bound $C_{s,mac}^{f-in-1}$ on $C_{s,mac}^{f}$ is in fact the capacity region $C_{mac}$ of the AWGN MAC without feedback, i.e.,*

(6) $$\begin{array}{cc} \; & C_{s,mac}^{f-in-1}=C_{mac}=\left\{\left(R_{1},R_{2}\right):R_{1}\leq \frac{1}{2}\log\left(1+\frac{P_{1}}{\sigma^{2}}\right),\right.\\ \; & \left.R_{2}\leq \frac{1}{2}\log\left(1+\frac{P_{2}}{\sigma^{2}}\right),\;\;R_{1}+R_{2}\leq \frac{1}{2}\log\left(1+\frac{P_{1}+P_{2}}{\sigma^{2}}\right)\right\}. \end{array}$$

*Comparing (5) and (6), we conclude that the secret key scheme is not optimal for the AWGN-MAC-E-CF. In the next section, we propose a new feedback scheme that achieves $C_{s,mac}^{f}$ in Theorem 1.*

### 2.3. Numerical Example

**Figure 2:**
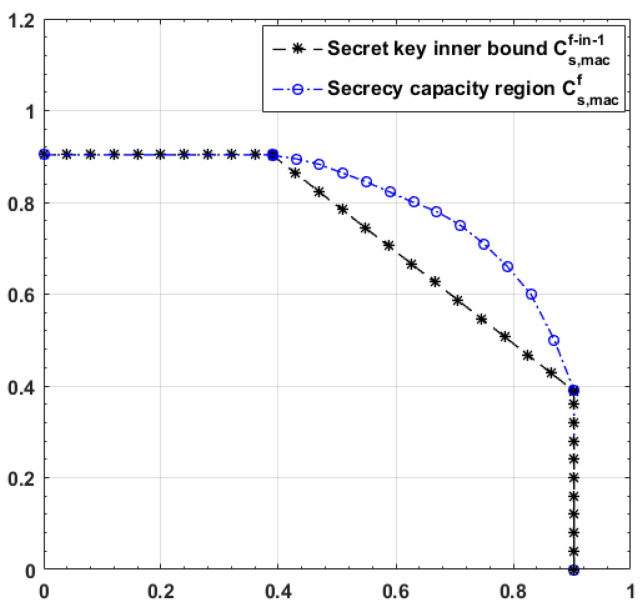
Capacity results on the AWGN-MAC-E-CF, where $P_{1}=5$, $P_{2}=5$, $\sigma^{2}=5$, $\sigma_{e}^{2}=5$.

The following Figure 2 plots $C_{s,mac}^{f}$ and $C_{s,mac}^{f-in-1}$ for $P_{1}=5$, $P_{2}=5$, $\sigma^{2}=5$, $\sigma_{e}^{2}=5$. It is easy to see that the gap is obvious and the secret key feedback scheme is not optimal for the AWGN-MAC-E-CF.

## 3. Proof of the Theorem 1

**Figure 3:**
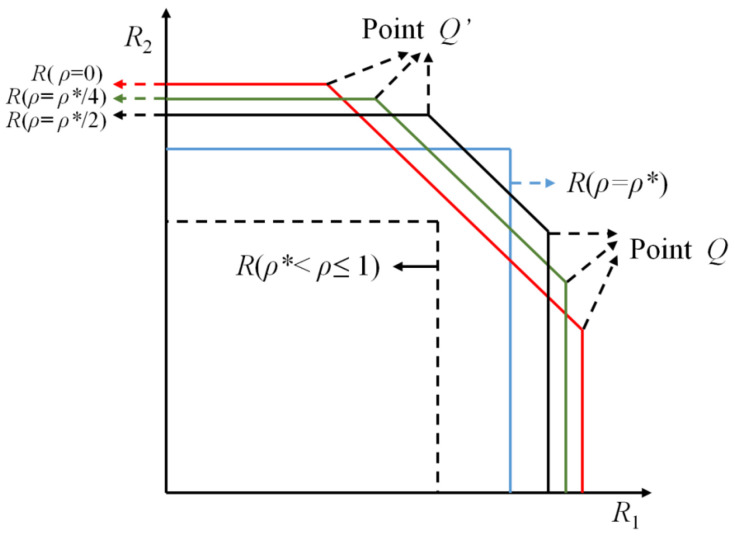
Illustration of R(ρ) for 0≤ρ≤1.

First, note that $C_{s,mac}^{f}$ cannot exceed the capacity region of the same model without the secrecy constraint, i.e., $C_{s,mac}^{f}\subseteq C_{mac}^{f}$. Then, it remains to be proven that any rate pair $\left(R_{1},R_{2}\right)\in C_{mac}^{f}$ is achievable with PWS defined in (4), which is equivalent to show that for any 0≤ρ≤1, R(ρ) in (5) is achievable with PWS. In Figure 3, we plot R(ρ) for all 0≤ρ≤1, where $\rho^{*}$ is the ρ satisfying the sum of the right hand side (RHS) of the first two inequalities in (5), which equals the RHS of the third inequality, which is equivalent to $\rho^{*}$ (the solution in (0,1)), of

(7) $$\begin{array}{cc} \; & \sigma^{2}\left(\sigma^{2}+P_{1}+P_{2}+2\sqrt{P_{1}P_{2}}\rho \right)=\left[\sigma^{2}+P_{1}\left(1-\rho^{2}\right)\right]\left[\sigma^{2}+P_{2}\left(1-\rho^{2}\right)\right]. \end{array}$$

From Figure 3, we see that when $\rho^{*}<\rho \leq 1$, R(ρ) is included in $R(\rho^{*})$, hence we only need to prove that for any $0\leq \rho \leq \rho^{*}$, R(ρ) is achievable with PWS. In the remainder of this section, the proof is given by two cases, i.e., $\rho =\rho^{*}$ and $0\leq \rho <\rho^{*}$. The details are given below.

### 3.1. Case 1: $\rho =\rho^{*}$

In this case, we directly show that Ozarow’s SK-type feedback scheme for AWGN MAC with feedback [8] is achievable with PWS. The basic intuition behind this scheme is described below. First, recall that for the classical point-to-point SK scheme, the receiver estimates the transmitted message by minimum mean square estimation (MMSE), and through a noiseless feedback channel, the estimation error of the receiver’s estimation is known by the transmitter since he knows the real transmitted message, and hence in the next time, the transmitter encodes this estimation error as a codeword and sends it to the receiver with the AWGN channel. By iteration, the receiver’s estimation error vanishes as the coding blocklength tends to infinity. Then, for the two-user AWGN MAC with noiseless feedback, by viewing each other’s transmitted codeword as part of the channel noise, this MAC model can be equivalent to two point-to-point AWGN channels with noiseless feedback. In addition to this, to further increase the sum rate of this MAC model, a modulation factor ρ is applied to the second user’s encoder, which helps to enhance the mutual information between the transceiver. The detail of this scheme is given below.

For k∈{1,2}, let $W_{k}=\left\{1,2,\ldots ,2^{NR_{k}}\right\}$ be the message set of $W_{k}$, divide the interval [−0.5,0.5] into $2^{NR_{k}}$ equally spaced sub-intervals, and each sub-interval center is mapped to a value in $W_{k}$. The center of the sub-interval with respect to (w.r.t.) $W_{k}$ is denoted by $\Theta_{k}$, where its variance approximately equals $\frac{1}{12}$.

*Coding procedure*:

At time instant 1, Transmitter 2 sends nothing but zero, i.e., $X_{2,1}=0$, and Transmitter 1 sends

(8) $$X_{1,1}=\sqrt{12P_{1}}\Theta_{1}+S,$$

where $S∼N(0,\sigma_{0}^{2})$ is a Gaussian random variable and it is independent of the transmitted message and all signals in Figure 1. Here, *S* is used to obtain a steady $\rho_{i}$ for 2≤i≤N, and this will be explained later.

Once the legal receiver obtains $Y_{1}=\sqrt{12P_{1}}\Theta_{1}+S+\eta_{1}$, his first estimation ${\hat{\Theta }}_{1,1}$ about $\Theta_{1}$ is given by

(9) $${\hat{\Theta }}_{1,1}=\frac{Y_{1}}{\sqrt{12P_{1}}}=\Theta_{1}+\frac{S+\eta_{1}}{\sqrt{12P_{1}}}.$$

For continuity, define the legal receiver’s first estimation of $\Theta_{2}$ as ${\hat{\Theta }}_{2,1}=0$.

At the end of time instant 1, Transmitter 1 receives $Y_{1}$ via channel feedback, and he computes the error $\epsilon_{1,1}$ of the legal receiver’s first estimation about $\Theta_{1}$ by

(10) $$\epsilon_{1,1}={\hat{\Theta }}_{1,1}-\Theta_{1}=\frac{S+\eta_{1}}{\sqrt{12P_{1}}},$$

where the variance of $\epsilon_{1,1}$ is given by

(11) $$\alpha_{1,1}=Var\left(\epsilon_{1,1}\right)=E\left[{\left(\frac{S+\eta_{1}}{\sqrt{12P_{1}}}\right)}^{2}\right]=\frac{\sigma_{0}^{2}+\sigma^{2}}{12P_{1}}.$$

At time instant 2, Transmitter 1 sends nothing but zero, i.e., $X_{1,2}=0$, and Transmitter 2 sends

(12) $$X_{2,2}=\sqrt{12P_{2}}\Theta_{2}+S.$$

Once the legal receiver obtains $Y_{2}=\sqrt{12P_{2}}\Theta +S+\eta_{2}$, his second estimation ${\hat{\Theta }}_{2,2}$ about $\Theta_{2}$ is given by

(13) $${\hat{\Theta }}_{2,2}=\frac{Y_{2}}{\sqrt{12P_{2}}}=\Theta_{2}+\frac{S+\eta_{2}}{\sqrt{12P_{2}}}.$$

For continuity, define the legal receiver’s second estimation of $\Theta_{1}$ as ${\hat{\Theta }}_{1,2}={\hat{\Theta }}_{1,1}$, which indicates that $\epsilon_{1,2}=\epsilon_{1,1}$, and $\alpha_{1,2}=\alpha_{1,1}$.

At the end of time instant 2, Transmitter 2 receives $Y_{2}$ via channel feedback, and computes the error $\epsilon_{2,2}$ of the legal receiver’s second estimation about $\Theta_{2}$ by

(14) $$\epsilon_{2,2}={\hat{\Theta }}_{2,2}-\Theta_{2}=\frac{S+\eta_{2}}{\sqrt{12P_{2}}},$$

where the variance of $\epsilon_{2,2}$ is given by

(15) $$\alpha_{2,2}=Var\left(\epsilon_{2,2}\right)=E\left[{\left(\frac{S+\eta_{2}}{\sqrt{12P_{2}}}\right)}^{2}\right]=\frac{\sigma_{0}^{2}+\sigma^{2}}{12P_{2}}.$$

At time instant 3≤i≤N, first, define

(16) $$\rho_{i-1}=\frac{E[\epsilon_{1,i-1}\epsilon_{2,i-1}]}{\sqrt{\alpha_{1,i-1}\alpha_{2,i-1}}}$$

as the correlation coefficient of $\epsilon_{1,i-1}$ and $\epsilon_{2,i-1}$, which are the legal receiver’s estimation errors of $\Theta_{1}$ and $\Theta_{2}$ at the time instant i−1. Moreover, note that $\alpha_{k,i-1}$ (k∈{1,2}) is the variance of $\epsilon_{k,i-1}$. Next, define the symbolic function $\mathrm{sgn}(\rho_{i-1})$ of $\rho_{i-1}$ as

(17) $$\mathrm{sgn}\left(\rho_{i-1}\right)=\left\{\begin{array}{cc} \;1, & \;\;\;\rho_{i-1}\geq 0\\ \;-1, & \;\;\;\rho_{i-1}<0 \end{array}\right.$$

which is used as a modulation factor maximizing the mutual information between the transmitters and the legal receiver. Then, Transmitters 1 and 2 send

(18) $$\begin{array}{cc} \; & X_{1,i}=\sqrt{\frac{P_{1}}{\alpha_{1,i-1}}}\epsilon_{1,i-1},\;\;\;X_{2,i}=\sqrt{\frac{P_{2}}{\alpha_{2,i-1}}}\epsilon_{2,i-1}\cdot \mathrm{sgn}\left(\rho_{i-1}\right),respectively. \end{array}$$

Once receiving $Y_{i}=X_{1,i}+X_{2,i}+\eta_{i}$, the legal receiver updates his estimation of $\Theta_{k}$ by

(19) $${\hat{\Theta }}_{k,i}={\hat{\Theta }}_{k,i-1}-\beta_{k,i}Y_{i},$$

where

(20) $$\beta_{k,i}=\frac{E[\epsilon_{k,i-1}Y_{i}]}{E[Y_{i}^{2}]}.$$

Since $\epsilon_{k,i}={\hat{\Theta }}_{k,i}-\Theta_{k}$, (19) can be rewritten as

(21) $$\epsilon_{k,i}=\epsilon_{k,i-1}-\beta_{k,i}Y_{i}.$$

For 3≤i≤N, the variance $\alpha_{k,i}$(k∈{1,2}) of $\epsilon_{k,i}$ can be calculated as

(22) $$\alpha_{k,i}=\alpha_{k,i-1}\frac{\sigma^{2}+P_{k}\left(1-\rho_{i-1}^{2}\right)}{P_{1}+P_{2}+2\sqrt{P_{1}P_{2}}\left|\rho_{i-1}\right|+\sigma^{2}}.$$

Now, substituting (21) and (22) into (16), we have

(23) $$\rho_{i}=\frac{\rho_{i-1}\sigma^{2}-\mathrm{sgn}\left(\rho_{k-1}\right)\sqrt{P_{1}P_{2}}\left(1-\rho_{i-1}^{2}\right)}{\sqrt{\left[P_{1}\left(1-\rho_{i-1}^{2}\right)+\sigma^{2}\right]\left[P_{2}\left(1-\rho_{i-1}^{2}\right)+\sigma^{2}\right]}},$$

where

(24) $$\rho_{2}=\frac{\sigma_{0}^{2}}{\sigma_{0}^{2}+\sigma^{2}}.$$

In general, $|\rho_{i}\left|\neq \right|\rho_{i-1}|$, to find a steady point in $|\rho_{i}|$, i.e., $|\rho_{i}\left|=\right|\rho_{i-1}|$, we substitute (23) into $1-\rho_{i}^{2}=1-\rho_{i-1}^{2}$, which is equivalent to

(25) $$\begin{array}{cc} \; & \sigma^{2}\left(\sigma^{2}+P_{1}+P_{2}+2\sqrt{P_{1}P_{2}}\rho_{i-1}\right)=\left[\sigma^{2}+P_{1}\left(1-\rho_{i-1}^{2}\right)\right]\left[\sigma^{2}+P_{2}\left(1-\rho_{i-1}^{2}\right)\right]. \end{array}$$

Here, note that the equation in (25) is exactly the same as that in (7), and $\rho^{*}$ is the solution to this equation. Hence, choosing an appropriate variance $\sigma_{0}^{2}$ of *S* such that $\rho_{2}$ in (24) satisfies $\rho_{2}=\rho^{*}$, we conclude that $|\rho_{i}|=\rho^{*}$ for all 2≤i≤N.

Next, following the error probability analysis in [8], we conclude that if $\left(R_{1},R_{2}\right)\in R\left(\rho^{*}\right)$, $P_{e}\to 0$, as N→∞. Now it remains to show that any rate pair $\left(R_{1},R_{2}\right)\in R\left(\rho^{*}\right)$ satisfies PWS; see the details below.

*Equivocation analysis*: first, note that for 3≤i≤N, the codewords $X_{1,i}$ and $X_{2,i}$ are linear combinations of $\eta_{1},\ldots ,\eta_{i-1}$, and *S*, which is in parallel to that of the classical SK scheme [9] for the point-to-point AWGN channel. Then the eavesdropper’s equivocation rate is bounded by

(26) $$\begin{array}{cc} \; & \Delta =\frac{1}{N}H\left(W_{1},W_{2}|Z^{N},{\tilde{Z}}^{N-1}\right)\overset{\left(a\right)}{=}\frac{1}{N}H\left(\Theta_{1},\Theta_{2}|Z^{N},{\tilde{Z}}^{N-1}\right)\\ \; & \geq \frac{1}{N}H\left(\Theta_{1},\Theta_{2}|Z^{N},{\tilde{Z}}^{N-1},\eta_{1},\ldots ,\eta_{N},S\right)\\ \; & =\frac{1}{N}H\left(\Theta_{1},\Theta_{2}\right|\underset{Z_{1}}{\underbrace{\sqrt{12P_{1}}\Theta_{1}+S+\eta_{e,1}}},\underset{Z_{2}}{\underbrace{\sqrt{12P_{2}}\Theta_{2}+S+\eta_{e,2}}},\underset{Z_{3},\ldots ,Z_{N}}{\underbrace{X_{1,3}+X_{2,3}+\eta_{e,3},\ldots ,X_{1,N}+X_{2,N}+\eta_{e,N}}},\eta_{1},\ldots ,\eta_{N},S,\\ \; & \underset{{\tilde{Z}}_{1}}{\underbrace{\sqrt{12P_{1}}\Theta_{1}+S+\eta_{1}+\eta_{d,1}}},\underset{{\tilde{Z}}_{2}}{\underbrace{\sqrt{12P_{2}}\Theta_{2}+S+\eta_{2}+\eta_{d,2}}},\underset{{\tilde{Z}}_{3},\ldots ,{\tilde{Z}}_{N-1}}{\underbrace{X_{1,3}+X_{2,3}+\eta_{3}+\eta_{d,3},\ldots ,X_{1,N-1}+X_{2,N-1}+\eta_{N-1}+\eta_{d,N-1}}})\\ \; & \overset{\left(b\right)}{=}\frac{1}{N}H\left(\Theta_{1},\Theta_{2}\right|\sqrt{12P_{1}}\Theta_{1}+\eta_{e,1},\sqrt{12P_{2}}\Theta_{2}+\eta_{e,2},\sqrt{12P_{1}}\Theta_{1}+\eta_{d,1},\sqrt{12P_{2}}\Theta_{2}+\eta_{d,2},\\ \; & \eta_{e,3},\ldots ,\eta_{e,N},\eta_{d,3},\ldots ,\eta_{d,N-1},\eta_{1},\ldots ,\eta_{N},S)\\ \; & \overset{\left(c\right)}{=}\frac{1}{N}H\left(\Theta_{1},\Theta_{2}|\sqrt{12P_{1}}\Theta_{1}+\eta_{e,1},\sqrt{12P_{2}}\Theta_{2}+\eta_{e,2},\sqrt{12P_{1}}\Theta_{1}+\eta_{d,1},\sqrt{12P_{2}}\Theta_{2}+\eta_{d,2}\right)\\ \; & \overset{\left(d\right)}{\geq }\frac{H\left(\Theta_{1}\right)+H\left(\Theta_{2}\right)+h\left(\eta_{d,1}\right)+h\left(\eta_{d,2}\right)+h\left(\eta_{e,1}\right)+h\left(\eta_{e,2}\right)}{N}-\frac{h\left(\sqrt{12P_{1}}\Theta_{1}+\eta_{e,1}\right)+h\left(\sqrt{12P_{2}}\Theta_{2}+\eta_{e,2}\right)}{N}\\ \; & -\frac{h\left(\sqrt{12P_{1}}\Theta_{1}+\eta_{d,1}\right)+h\left(\sqrt{12P_{2}}\Theta_{2}+\eta_{d,2}\right)}{N}\\ \; & \overset{\left(e\right)}{\geq }R_{1}+R_{2}-\frac{1}{N}\underset{\mathrm{information}\;\mathrm{leakage}\;\mathrm{on}\;\mathrm{the}\;\mathrm{forward}\;\mathrm{channel}}{\underbrace{\left[\frac{1}{2}\log\left(1+\frac{P_{1}}{\sigma_{e}^{2}}\right)+\frac{1}{2}\log\left(1+\frac{P_{2}}{\sigma_{e}^{2}}\right)\right]}}-\frac{1}{N}\underset{\mathrm{information}\;\mathrm{leakage}\;\mathrm{on}\;\mathrm{the}\;\mathrm{feedback}\;\mathrm{channel}}{\underbrace{\left[\frac{1}{2}\log\left(1+\frac{P_{1}}{\sigma_{d}^{2}}\right)+\frac{1}{2}\log\left(1+\frac{P_{2}}{\sigma_{d}^{2}}\right)\right]}}, \end{array}$$

where (a) follows from the fact that $\Theta_{k}$ (k=1,2) is a deterministic function of $W_{k}$, (b) follows from the fact that $X_{1,i}$ and $X_{2,i}$ are linear combinations of $\eta_{1},\ldots ,\eta_{i-1}$, and *S*, (c) follows from the fact that $\Theta_{1}$, $\Theta_{2}$, $\eta_{e,1}$, $\eta_{e,2}$, $\eta_{d,1}$, $\eta_{d,2}$ are independent of $\eta_{e,3},\ldots ,\eta_{e,N}$, $\eta_{1}$,…, $\eta_{N}$, $\eta_{d,3},\ldots ,\eta_{d,N}$, and *S*, (d) follows from the fact that $\Theta_{1}$, $\Theta_{2}$, $\eta_{e,1}$, $\eta_{e,2}$, $\eta_{d,1}$, $\eta_{d,2}$ are independent of one another, (e) follows from $H\left(\Theta_{1}\right)=NR_{1}$, $H\left(\Theta_{2}\right)=NR_{2}$, and the variance of $\Theta_{k}$ (k=1,2) equals $\frac{1}{12}$ as *N* tends to infinity.

From (26), we conclude that choosing sufficiently large *N*, the secrecy constraint $\Delta =\frac{H(W_{1},W_{2}|Z^{N},{\tilde{Z}}^{N-1})}{N}\geq R_{1}+R_{2}-\epsilon$ in (4) is guaranteed, which indicates that any pair $\left(R_{1},R_{2}\right)$ in $R(\rho^{*})$ is achievable with PWS.

### 3.2. Case 2: $0\leq \rho <\rho^{*}$

In this case, we show that the pentagon rate region $R(0\leq \rho <\rho^{*})$ in Figure 3 is achievable with PWS. We only need to show that the corner point *Q* is achievable with PWS, then by symmetry, $Q^{\prime }$ is also achievable with PWS, finally, using time sharing between *Q* and $Q^{\prime }$, the line $QQ^{\prime }$ is achievable with PWS, which indicates that the entire region $R(0\leq \rho <\rho^{*})$ is achievable with PWS. The secure coding scheme that achieves *Q* is briefly explained below. Divide the message $W_{1}$ of Transmitter 1 into two parts, where one part together with the message $W_{2}$ of Transmitter 2 are encoded by the SK-type scheme shown in case 1, and the other part of $W_{1}$ is encrypted by a key which is generated by the channel noise at the first time instant and this key is only known by the legal parties. In case 1 we have shown that Ozarow’s SK-type scheme is achievable with PWS, and note that the other part of $W_{1}$ is also achievable with PWS since it is protected by a secret key, which indicates that the whole scheme satisfies PWS. The details of our proposed scheme are given below.

*Message splitting*: the message $W_{1}$ is divided into two independent parts $\left(W_{a},W_{b}\right)$, where $W_{a}$ takes values in $W_{a}=\left\{1,2,\ldots ,2^{NR_{a}}\right\}$, $W_{b}$ takes values in $W_{b}=\left\{1,2,\ldots ,2^{NR_{b}}\right\}$, and $R_{a}+R_{b}=R_{1}$. $W_{2}$ takes values in $W_{2}=\left\{1,2,\ldots ,2^{NR_{2}}\right\}$. Divide the interval [−0.5,0.5] into $2^{NR_{l}}\left(l\in \left\{b,2\right\}\right)$ equally spaced sub-intervals, and each sub-interval center corresponds to a value in $W_{l}$. The center of the sub-interval w.r.t. $W_{b}$ ($W_{2}$) is denoted by $\Theta_{1}$ ($\Theta_{2}$), where the variance of $\Theta_{k}$ (k∈{1,2}) approximately equals $\frac{1}{12}$.

*Secret key generation*: at time instant 1, Transmitters 1 and 2 send $X_{1,1}=X_{2,1}=0$. The legal receiver receives $Y_{1}=X_{1,1}+X_{2,1}+\eta_{1}=\eta_{1}$, and transmits $Y_{1}$ back to the transmitters. Since $Y_{1}$ is continuous, we can generate a secret key *K* with arbitrary rate from $Y_{1}$ and this key is uniformly distributed in $W_{a}=\left\{1,2,\ldots ,2^{NR_{a}}\right\}$.

*Encoding-decoding procedure*: at time instants 2 and 3, the transmission codewords are exactly the same as those in case 1 at time instants 1 and 2, namely, $X_{1,2}=\sqrt{12P_{1}}\Theta_{1}+S$, $X_{2,2}=0$, $X_{1,3}=0$ and $X_{2,3}=\sqrt{12P_{2}}\Theta_{2}+S$.

At time instant 4≤i≤N, Transmitters 1 and 2 send

(27) $$\begin{array}{cc} \; & X_{1,i}=U_{i}+V_{i}=U_{i}+\sqrt{\frac{\left(1-\gamma \right)P_{1}}{\alpha_{1,i-1}}}\epsilon_{1,i-1},X_{2,i}=\sqrt{\frac{P_{2}}{\alpha_{2,i-1}}}\epsilon_{2,i-1}\mathrm{sgn}\left(\rho_{i-1}\right), \end{array}$$

respectively, where $U_{i}$ is the codeword of the encrypted sub-message $W_{a}⊕K$ with transmission power $\gamma P_{1}$(0≤γ≤1), $V_{i}$ is the codeword of the sub-message $W_{b}$ with transmission power $\left(1-\gamma \right)P_{1}$. Here, note that the codeword $U_{4}^{N}=\left(U_{4},\ldots ,U_{N}\right)$ is generated by Shannon’s random coding scheme [3], namely, each component of $U_{4}^{N}$ is i.i.d. generated according to the Gaussian distribution with zero mean and variance $\gamma P_{1}$, and $U_{4}^{N}$ is one-to-one mapped to a value of $W_{a}⊕K$. In addition, for 4≤i≤N, $V_{i}$ and $X_{2,i}$ (codewords for $W_{b}$ and $W_{2}$) are generated in the same way as the SK-type scheme of case 1, where $U_{i}+\eta_{i}$ is viewed as the “channel noise” for the codewords $V_{i}$ and $X_{2,i}$. Note that $\epsilon_{1,i}$, $\epsilon_{2,i}$, $\alpha_{1,i}$, $\alpha_{2,i}$, $\rho_{i}$, and $\mathrm{sgn}(\rho_{i})$ are defined in the same way as those in Section 3.1 by replacing $\eta_{i}$ by $U_{i}+\eta_{i}$.

*Decoding procedure*: successive cancellation decoding is employed, specifically, first, viewing $U_{i}+\eta_{i}$ as the equivalent channel noise and using the SK-type decoding scheme in case 1, for sufficiently large *N*, $W_{b}$ and $W_{2}$ can be decoded by the legal receiver with arbitrary small decoding error probability if

(28) $$\begin{array}{cc} \; & R_{b}\leq \frac{1}{2}\log\left[1+\frac{\left(1-\gamma \right)P_{1}}{\sigma^{2}+\gamma P_{1}}\left(1-\rho^{**2}\right)\right],\\ \; & R_{2}\leq \frac{1}{2}\log\left[1+\frac{P_{2}}{\sigma^{2}+\gamma P_{1}}\left(1-\rho^{**2}\right)\right], \end{array}$$

where $\rho^{**}$ is the solution in (0,1) of

(29) $$\begin{array}{cc} \; & \left(\sigma^{2}+\gamma P_{1}\right)\left[\sigma^{2}+\gamma P_{1}+\left(1-\gamma \right)P_{1}+P_{2}+2\sqrt{\left(1-\gamma \right)P_{1}P_{2}}\rho^{**}\right]\\ \; & =\left[\sigma^{2}+\gamma P_{1}+\left(1-\gamma \right)P_{1}\left(1-\rho^{**2}\right)\right]\left[\sigma^{2}+\gamma P_{1}+P_{2}\left(1-\rho^{**2}\right)\right]. \end{array}$$

Here, note that $\frac{N}{N-1}R_{b}$ and $\frac{N}{N-1}R_{2}$ are actual transmission rates of $W_{b}$ and $W_{2}$, respectively. For sufficiently large *N*, $\frac{N}{N-1}R_{b}$ and $\frac{N}{N-1}R_{2}$ tend to $R_{b}$ and $R_{2}$, respectively.

After decoding $W_{b}$ and $W_{2}$, the legal receiver subtracts $V_{i}$ and $X_{2,i}$ from his received signal $Y_{i}$, which indicates that the channel noise of the equivalent channel for the transmission of $U_{i}$ is $\eta_{i}$, then based on the channel coding theorem [3], we conclude that for sufficiently large *N*, $W_{a}$ can be decoded by the legal receiver with arbitrary small decoding error probability if

(30) $$R_{a}\leq \frac{1}{2}\log\left(1+\frac{\gamma P_{1}}{\sigma^{2}}\right).$$

Here, note that $\frac{N}{N-3}R_{b}$ is the actual transmission rate of $W_{a}$, and for sufficiently large *N*, $\frac{N}{N-3}R_{b}$ tends to $R_{a}$.

From (28) and (30), $R_{1}=R_{a}+R_{b}$, and letting $\rho =\sqrt{\left(1-\gamma \right)}\rho^{**}$, we conclude that any pair $\left(R_{1},R_{2}\right)$ in $R(0\leq \rho <\rho^{*})$ is achievable. Now it remains to be shown that any rate pair $\left(R_{1},R_{2}\right)\in R\left(0\leq \rho <\rho^{*}\right)$ satisfies PWS; see the details below.

*Equivocation analysis*: the eavesdropper’s equivocation rate is bounded by

(31) $$\begin{array}{cc} \frac{H(W_{1},W_{2}|Z^{N},{\tilde{Z}}^{N-1})}{N} & =\frac{H(W_{a},W_{b},W_{2}|Z^{N},{\tilde{Z}}^{N-1})}{N}\\ \; & =\frac{H(W_{a}|Z^{N},{\tilde{Z}}^{N-1})}{N}+\frac{H(W_{b},W_{2}|W_{a},Z^{N},{\tilde{Z}}^{N-1})}{N}. \end{array}$$

The first term in (31) can be calculated by

(32) $$\begin{array}{cc} \frac{H(W_{a}|Z^{N},{\tilde{Z}}^{N-1})}{N} & \geq \frac{H(W_{a}|Z^{N},{\tilde{Z}}^{N-1},U_{4}^{N})}{N}\overset{\left(a\right)}{=}\frac{H(W_{a}|U_{4}^{N})}{N}\overset{\left(b\right)}{=}\frac{H(W_{a}|U_{4}^{N},W_{a}⊕K)}{N}\\ \; & =\frac{H(K|U_{4}^{N},W_{a}⊕K)}{N}\overset{\left(c\right)}{=}\frac{H(K)}{N}=R_{a}, \end{array}$$

where (a) follows from the Markov chain $W_{a}\to U_{4}^{N}\to \left(Z^{N},{\tilde{Z}}^{N-1}\right)$, (b) follows from $U_{4}^{N}$ is a deterministic function of $\left(W_{a}⊕K\right)$, and (c) follows from *K* is independent of $\left(W_{a}⊕K\right)$ and $U_{4}^{N}$, and *K* is uniformly drawn from $\left\{1,2,\ldots ,2^{NR_{a}}\right\}$.

For the second term in (31), along the lines of the equivocation analysis in case 1, we conclude that

(33) $$\begin{array}{cc} \; & \frac{1}{N}H\left(W_{b},W_{2}|Z^{N},{\tilde{Z}}^{N-1},W_{a}\right)=\frac{1}{N}H\left(W_{b},W_{2}|Z^{N},{\tilde{Z}}^{N-1},W_{a},U_{4}^{N}\right)\\ \; & \geq R_{b}+R_{2}-\frac{1}{2N}\log\left(1+\frac{P_{1}}{\sigma_{e}^{2}}\right)-\frac{1}{2N}\log\left(1+\frac{P_{2}}{\sigma_{e}^{2}}\right)-\frac{1}{2N}\log\left(1+\frac{P_{1}}{\sigma_{d}^{2}}\right)-\frac{1}{2N}\log\left(1+\frac{P_{2}}{\sigma_{d}^{2}}\right). \end{array}$$

Substituting (32) and (33) into (31), choosing sufficiently large *N*, $\Delta =\frac{H(W_{1},W_{2}|Z^{N},{\tilde{Z}}^{N-1})}{N}\geq R_{1}+R_{2}-\epsilon$ is guaranteed, which completes the proof.

## 4. Discussion

In this section, we show that Ozarow’s scheme is in fact a secure finite blocklength (FBL) coding scheme, and characterize its sum rate under fixed coding blocklength, decoding error probability and the eavesdropper’s uncertainty about the transmitted messages. Then, we further explain the results via numerical examples.

### 4.1. The Definition of the Secure FBL Scheme for the AWGN-MAC-E-CF

For the AWGN-MAC-E-CF, the channel’s input and output relationship is given in Section 2.1.

A $\left(N,|W_{1}|,|W_{2}|,P_{1},P_{2}\right)$-code under average power constraints consists of:

- Message $W_{k}\left(k\in \left\{1,2\right\}\right)$, uniformly drawn in $W_{k}=\left\{1,2,\ldots ,\right|W_{k}\left|\right\}$.
- Encoder *k* with outputs $X_{k,i}=f_{k,i}\left(W_{k},Y_{k,1}^{i-1}\right)$ satisfies the average power constraints
  (34) $$\frac{1}{N}\sum_{i=1}^{N}E\left[X_{k,i}^{2}\right]\leq P_{k},$$
  where $f_{k,i}\left(\cdot \right)$ is a (stochastic) function.
- The decoder with outputs
  (35) $$\left({\hat{W}}_{1},{\hat{W}}_{2}\right)=\psi \left(Y^{N}\right),$$
  where ψ is the decoding function of the Receiver.

The average decoding error probability $P_{ek}$ is defined as

(36) $$\begin{array}{cc} \; & P_{e}=Pr\left[\left({\hat{W}}_{1},{\hat{W}}_{2}\right)\neq \left(W_{1},W_{2}\right)\right]=\frac{\sum_{w_{1},w_{2}\in W_{1},W_{2}}Pr\left.\{\psi \left(Y^{N}\right)\neq \left(w_{1},w_{2}\right)|\left(W_{1},W_{2}\right)=\left(w_{1},w_{2}\right)\right.\}}{|W_{1}\left|\right|W_{2}|}. \end{array}$$

In addition, define the eavesdropper’s normalized equivocation (also called the secrecy level) as

(37) $$\Delta_{f}=\frac{H(W_{1},W_{2}|Z^{N},{\tilde{Z}}^{N-1})}{H(W_{1},W_{2})},$$

where 0≤Δ≤1. The (N,ϵ,δ)-rate pair $\left(R_{1}\left(N,\epsilon ,\delta \right),R_{2}\left(N,\epsilon ,\delta \right)\right)$ is achievable with a secrecy level of δ(0≤δ≤1) if for given blocklength *N*, error probability ϵ and secrecy level δ, there exists a $\left(N,|W_{1}|,|W_{2}|,P_{1},P_{2}\right)$-code described above such that

(38) $$\frac{\log|W_{1}|}{N}=R_{1}\left(N,\epsilon ,\delta \right),\;\;\;\frac{\log|W_{2}|}{N}=R_{2}\left(N,\epsilon ,\delta \right),\;\;\;P_{e}\leq \epsilon ,\;\;\;\Delta_{f}\geq \delta .$$

For the AWGN-MAC-E-CF, the achievable sum-rate is denoted by

(39) $$R_{sum}\left(N,\epsilon ,\delta \right)=R_{1}\left(N,\epsilon ,\delta \right)+R_{2}\left(N,\epsilon ,\delta \right),$$

and the maximal sum-rate $R_{sum}^{*}\left(N,\epsilon ,\delta \right)$ is the maximum sum-rate $R_{sum}\left(N,\epsilon ,\delta \right)$ defined in (39).

### 4.2. Main Result

**Theorem 2.**  *For given decoding error probability ϵ and boding blocklength N, let $R_{sum}\left(N,\epsilon \right)$ be the achievable sum-rate of the SK-type scheme for the AWGN-MAC-E-CF without the consideration of secrecy. Then for a given secrecy level δ, if the coding blocklength N in $R_{sum}\left(N,\epsilon \right)$ satisfies*

(40) $$NR_{sum}\left(N,\epsilon \right)\geq \frac{1}{2(1-\delta )}\log\left[\left(1+\frac{P_{1}}{\sigma_{e}^{2}}\right)\left(1+\frac{P_{2}}{\sigma_{e}^{2}}\right)\left(1+\frac{P_{1}}{\sigma_{d}^{2}}\right)\left(1+\frac{P_{2}}{\sigma_{d}^{2}}\right)\right],$$

the rate $R_{sum}\left(N,\epsilon \right)$ also serves as a lower bound on the maximal sum-rate $R_{sum}^{*}\left(N,\epsilon ,\delta \right)$, i.e.,

(41) $$R_{sum}^{*}\left(N,\epsilon ,\delta \right)\geq R_{sum}\left(N,\epsilon \right),$$

*where*

(42) $$\begin{array}{cc} \; & R_{sum}\left(N,\epsilon \right)=\frac{1}{2}\log\left(1+\frac{P_{1}+P_{2}+2\rho^{*}\sqrt{P_{1}P_{2}}}{\sigma^{2}}\right)\\ \; & -\frac{1}{N}\log\left(\left(1+\frac{P_{1}+P_{2}+2\rho^{*}\sqrt{P_{1}P_{2}}}{\sigma^{2}}\right)\frac{\sigma_{0}^{2}+\sigma^{2}}{12\sqrt{P_{1}P_{2}}}{\left[Q^{-1}\left(\frac{\epsilon }{2}\right)\right]}^{2}\right), \end{array}$$

$\rho^{*}$ is the largest solution in (0,1) of

(43) $$\sigma^{2}\left(\sigma^{2}+P_{1}+P_{2}+2\sqrt{P_{1}P_{2}}\rho \right)=\left[\sigma^{2}+P_{1}\left(1-\rho^{2}\right)\right]\left[\sigma^{2}+P_{2}\left(1-\rho^{2}\right)\right],$$

*and $\sigma_{0}^{2}$ satisfies*

(44) $$\frac{\sigma_{0}^{2}}{\sigma_{0}^{2}+\sigma^{2}}=\rho^{*}.$$

**Proof.**  See Section 4.4. □

### 4.3. Numerical Results

**Figure 4:**
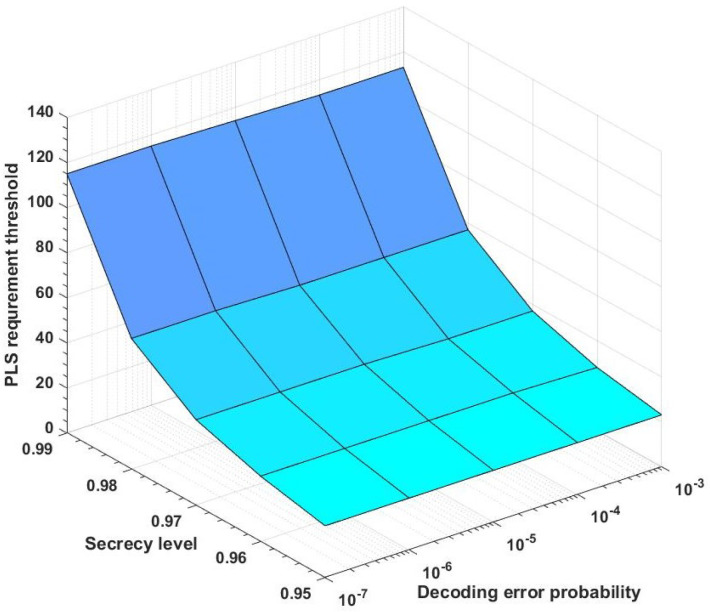
The relationship between secrecy level, decoding error probability, and PLS requirement blocklength threshold for the AWGN MAC with an external eavesdropper and feedback ($P_{1}=P_{2}=2$, $\sigma^{2}=1$).

Define the minimum blocklength *N* satisfying (40) as the PLS requirement blocklength threshold. Figure 4 plots the relationship between secrecy level, decoding error probability, and PLS requirement blocklength threshold for the AWGN MAC with an external eavesdropper and feedback ($P_{1}=P_{2}=2$, $\sigma^{2}=1$). From Figure 4, we conclude that for a fixed decoding error probability, the PLS requirement threshold is increasing while the secrecy level is increasing. Moreover, when the decoding error probability $\epsilon =10^{-7}$ and the secrecy level δ=0.99, the PLS requirement blocklength threshold is about 115.

**Figure 5:**
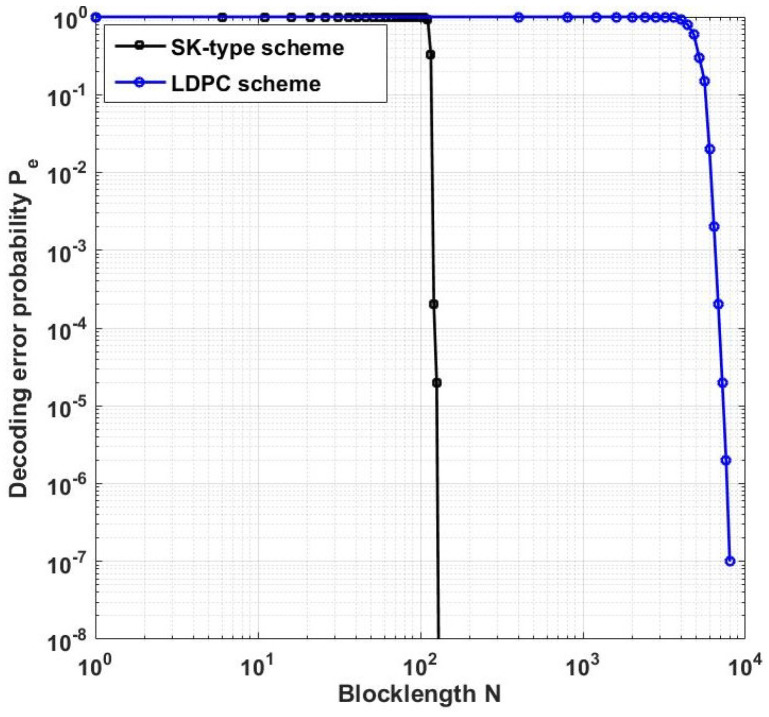
Comparison of the decoding error probability $P_{e}$ for $P_{1}=P_{2}=2$, $\sigma^{2}=1$ and *N* taking values in [0,8000].

Figure 5 plots the decoding error probability $P_{e}$ of Ozarow’s SK scheme [8] and LDPC code [11] for $P_{1}=P_{2}=2$, and the length of transmission bits is 80. From Figure 5, we conclude that compared with LDPC scheme, the average error probability $P_{e}$ of Ozarow’s SK scheme decays much faster with the increasing coding blocklength *N*.

### 4.4. Proof of the Theorem 2

*Encoding-decoding procudure:* in fact, Ozarow’s scheme [8] is inherently a secure FBL coding scheme. The encoding and decoding processes are exactly the same as those described in Section 3.1, so we omit the detailed explanation here.

*Decoding error probability analysis*: the target error probability of the whole scheme is chosen to be ϵ. Then, we let the error probability of transmitting $W_{k}$ be $P_{e,k}$ which at most ϵ/2, i.e.,

(45) $$P_{e,k}\leq \frac{\epsilon }{2}.$$

From (45) and the error probability analysis in [10], we have

(46) $$\begin{array}{cc} \; & R_{k}\left(N,\epsilon \right)=\frac{1}{2}\log\left[1+P_{k}\left(1-\rho^{*2}\right)\right]-\frac{1}{2N}\log\left({\left[1+P_{k}\left(1-\rho^{*2}\right)\right]}^{2}\frac{\sigma_{0}^{2}+\sigma_{k}^{2}}{12P_{k}}{\left[Q^{-1}\left(\frac{\epsilon }{2}\right)\right]}^{2}\right). \end{array}$$

Let $R_{k}\left(N,\epsilon \right)$ be message $W_{k}$’s achievable rate of the SK-type scheme for the AWGN-MAC-E-CF without the consideration of secrecy. From (39) and (46), we have $R_{sum}\left(N,\epsilon \right)$ which is given in (42).

*Equivocation analysis:* now we show the above scheme satisfies the PLS requirement when the coding blocklength is larger than a threshold.

(47) $$\begin{array}{cc} \; & \Delta_{f}=\frac{H(W_{1},W_{2}|Z^{N},{\tilde{Z}}^{N-1})}{H(W_{1},W_{2})}\geq \frac{H(W_{1},W_{2}|Z^{N},{\tilde{Z}}^{N-1},\eta_{1},\ldots ,\eta_{N},S)}{H(W_{1},W_{2})}\\ \; & \geq \frac{1}{H(W_{1},W_{2})}H\left(W_{1},W_{2}|Z^{N},{\tilde{Z}}^{N-1},\eta_{1},\ldots ,\eta_{N},S\right)\\ \; & =\frac{1}{H(W_{1},W_{2})}H\left(W_{1},W_{2}\right|\underset{Z_{1}}{\underbrace{\sqrt{12P_{1}}\Theta_{1}+S+\eta_{e,1}}},\underset{Z_{2}}{\underbrace{\sqrt{12P_{2}}\Theta_{2}+S+\eta_{e,2}}},\underset{Z_{3},\ldots ,Z_{N}}{\underbrace{X_{1,3}+X_{2,3}+\eta_{e,3},\ldots ,X_{1,N}+X_{2,N}+\eta_{e,N}}},\\ \; & \underset{{\tilde{Z}}_{1}}{\underbrace{\sqrt{12P_{1}}\Theta_{1}+S+\eta_{1}+\eta_{d,1}}},\underset{{\tilde{Z}}_{2}}{\underbrace{\sqrt{12P_{2}}\Theta_{2}+S+\eta_{2}+\eta_{d,2}}},\underset{{\tilde{Z}}_{3},\ldots ,{\tilde{Z}}_{N-1}}{\underbrace{X_{1,3}+X_{2,3}+\eta_{3}+\eta_{d,3},\ldots ,X_{1,N-1}+X_{2,N-1}+\eta_{N-1}+\eta_{d,N-1}}},\\ \; & \eta_{1},\ldots ,\eta_{N},S)\\ \; & \overset{\left(d\right)}{=}\frac{1}{H(W_{1},W_{2})}H\left(W_{1},W_{2}\right|\sqrt{12P_{1}}\Theta_{1}+\eta_{e,1},\sqrt{12P_{2}}\Theta_{2}+\eta_{e,2},\sqrt{12P_{1}}\Theta_{1}+\eta_{d,1},\sqrt{12P_{2}}\Theta_{2}+\eta_{d,2},\\ \; & \eta_{e,3},\ldots ,\eta_{e,N},\eta_{d,3},\ldots ,\eta_{d,N-1},\eta_{1},\ldots ,\eta_{N},S)\\ \; & \overset{\left(e\right)}{=}\frac{1}{H(W_{1},W_{2})}H\left(W_{1},W_{2}|\sqrt{12P_{1}}\Theta_{1}+\eta_{e,1},\sqrt{12P_{2}}\Theta_{2}+\eta_{e,2},\sqrt{12P_{1}}\Theta_{1}+\eta_{d,1},\sqrt{12P_{2}}\Theta_{2}+\eta_{d,2}\right)\\ \; & \overset{\left(f\right)}{\geq }\frac{H\left(W_{1},W_{2}\right)+h\left(\eta_{d,1}\right)+h\left(\eta_{d,2}\right)+h\left(\eta_{e,1}\right)+h\left(\eta_{e,2}\right)}{H(W_{1},W_{2})}-\frac{h\left(\sqrt{12P_{1}}\Theta_{1}+\eta_{e,1}\right)+h\left(\sqrt{12P_{2}}\Theta_{2}+\eta_{e,2}\right)}{H(W_{1},W_{2})}\\ \; & -\frac{h\left(\sqrt{12P_{1}}\Theta_{1}+\eta_{d,1}\right)+h\left(\sqrt{12P_{2}}\Theta_{2}+\eta_{d,2}\right)}{H(W_{1},W_{2})}\\ \; & \overset{\left(g\right)}{\geq }1-\frac{\log\left(1+\frac{P_{1}}{\sigma_{e}^{2}}\right)+\log\left(1+\frac{P_{2}}{\sigma_{e}^{2}}\right)+\log\left(1+\frac{P_{1}}{\sigma_{d}^{2}}\right)+\log\left(1+\frac{P_{2}}{\sigma_{d}^{2}}\right)}{2NR_{sum}\left(N,\epsilon \right)}, \end{array}$$

where (d) follows from the fact that $X_{1,i}$ and $X_{2,i}$ are linear combinations of $\eta_{1},\ldots ,\eta_{i-1}$, and *S*, (e) follows from the fact that $\Theta_{1}$, $\Theta_{2}$, $\eta_{e,1}$, $\eta_{e,2}$, $\eta_{d,1}$, $\eta_{d,2}$ are independent of $\eta_{e,3},\ldots ,\eta_{e,N}$, $\eta_{1}$,…, $\eta_{N}$, $\eta_{d,3},\ldots ,\eta_{d,N}$, and *S*, (f) follows from the fact that $W_{1}$, $W_{2}$, $\eta_{e,1}$, $\eta_{e,2}$, $\eta_{d,1}$, $\eta_{d,2}$ are independent of one another and the fact that $\Theta_{k}$ (k=1,2) is a deterministic function of $W_{k}$, (g) follows from the fact that $H\left(W_{1},W_{2}\right)=NR_{sum}\left(N,\epsilon \right)$($R_{sum}\left(N,\epsilon \right)$ is defined in Theorem 2), and the maximum differential entropy lemma [3]. Substituting (47) into (38), the secrecy constraint

(48) $$\Delta \geq 1-\frac{\log\left(1+\frac{P_{1}}{\sigma_{e}^{2}}\right)+\log\left(1+\frac{P_{2}}{\sigma_{e}^{2}}\right)+\log\left(1+\frac{P_{1}}{\sigma_{d}^{2}}\right)+\log\left(1+\frac{P_{2}}{\sigma_{d}^{2}}\right)}{2NR_{sum}\left(N,\epsilon \right)}\geq \delta$$

is guaranteed by choosing blocklength *N* such that

(49) $$NR_{sum}\left(N,\epsilon \right)\geq \frac{1}{2(1-\delta )}\log\left[\left(1+\frac{P_{1}}{\sigma_{e}^{2}}\right)\left(1+\frac{P_{2}}{\sigma_{e}^{2}}\right)\left(1+\frac{P_{1}}{\sigma_{d}^{2}}\right)\left(1+\frac{P_{2}}{\sigma_{d}^{2}}\right)\right].$$

The proof of Theorem 2 is completed.

## 5. Conclusions and Future Work

In this paper, we show that for the AWGN-MAC-E-CF, the traditional secret key feedback scheme is not optimal, and propose an optimal scheme that achieves the secrecy capacity region of the AWGN-MAC-E-CF, which combines the linear feedback coding scheme for the same model without the secrecy constraint and the secret key scheme. Possible future work could consist of checking whether this kind of hybrid scheme is still optimal for other multi-user AWGN channel models in the presence of an external eavesdropper and channel feedback.

## Data Availability

Not applicable.

## Abbreviations

The following abbreviations are used in this manuscript:

PLS	Physical layer security
AWGN	Additive white Gaussian noise
PWS	Perfect weak secrecy
MAC	Multiple-access channel
AWGN-MAC-E-CF	Multiple-access channel with an external eavesdropper and channel feedback
SK	Schalkwijk-Kailath
FBL	Finite blocklength
